# Supplementary material for: An Innovative Three-Stage Model for Prenatal Genetic Disorder Detection Based on Region-of-Interest in Fetal Ultrasound
Source: Bioengineering (Basel). 2023 Jul 23;10(7):873. doi: 10.3390/bioengineering10070873 (PMC10376765; doi:10.3390/bioengineering10070873)
Supplement: Supplementary file 1 [file bioengineering-10-00873-s001.zip › bioengineering-2444921-supplementary.pdf]

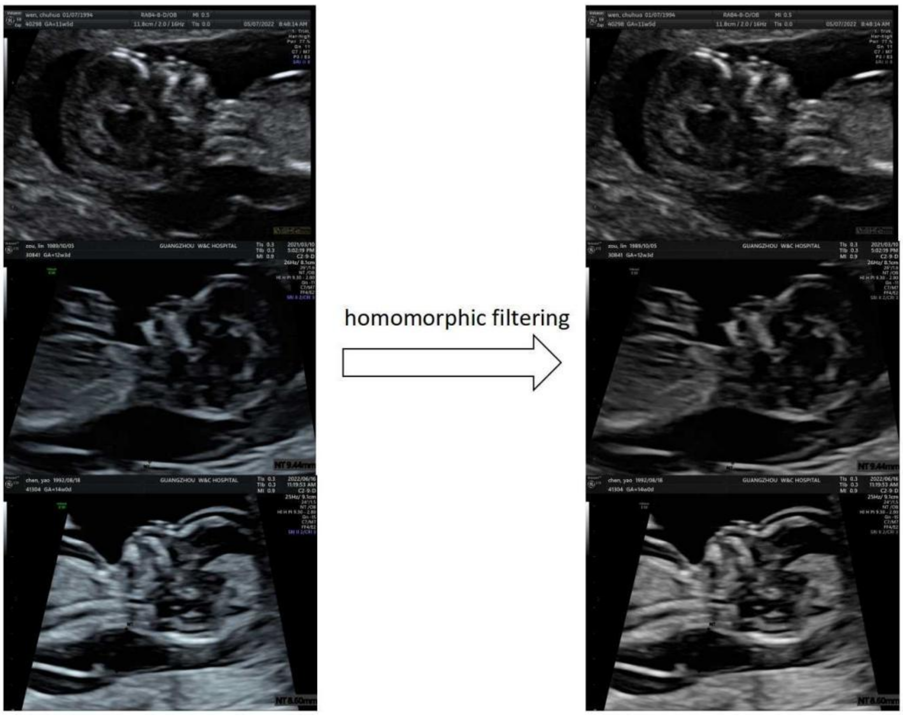

Supplementary Material S1. Example of homomorphic filtering results.

	NA+NB	Max+mand	Chin	Head2	Head1	NT
Model		CNN A			CNN B	
Conv1		7×7			3×3	
Conv2_x		$\begin{bmatrix}3\times 3,64\\3\times 3,64\\\text{CBAM}\end{bmatrix}\times 3$			$\begin{bmatrix}3\times 3,16\\3\times 3,16\\\text{CBAM}\end{bmatrix}\times 1$	
Conv3_x		$\begin{bmatrix}3\times 3,128\\3\times 3,128\\\text{CBAM}\end{bmatrix}\times 3$			$\begin{bmatrix}3\times 3,32\\3\times 3,32\\\text{CBAM}\end{bmatrix}\times 1$	
Conv4_x		$\begin{bmatrix}3\times 3,256\\3\times 3,256\\\text{CBAM}\end{bmatrix}\times 3$			$\begin{bmatrix}3\times 3,64\\3\times 3,64\\\text{CBAM}\end{bmatrix}\times 1$	
Conv5_x		$\begin{bmatrix}3\times 3,512\\3\times 3,512\\\text{CBAM}\end{bmatrix}\times 3$				
			Average pool			

Supplementary Material S2. The detailed structures of CNN A and CNN B. And the network structure can also be accessed at the following link: https://github.com/1057813680/modeling-chart/blob/main/FPMMM-34.png; https://github.com/1057813680/modeling-chart/blob/main/FPMMM-8.png

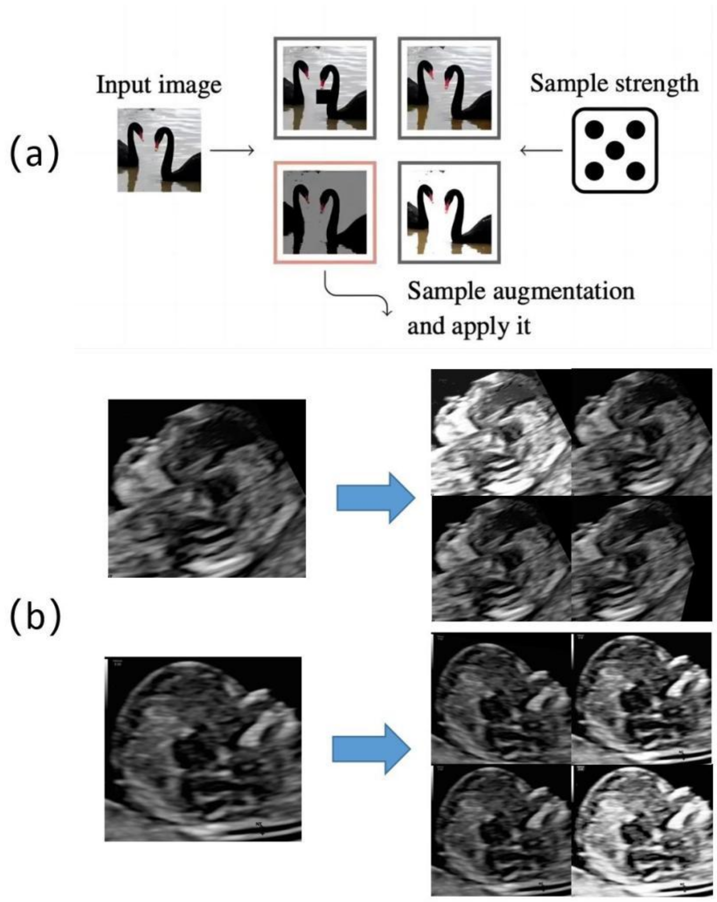

Supplementary Material S3. Example of Trivial Augment.
